# Computational modelling and sequence analysis provide new insights into the structure, function, and evolution of the *pirB* Gene in *Photorhabdus*, *Xenorhabdus*, and *Vibrio species*

**DOI:** 10.5455/javar.2025.l965

**Published:** 2025-09-23

**Authors:** Arren Christian M. de Guia, Mary Rose D. Uy-de Guia, Simon G. Alcantara, Claro N. Mingala

**Affiliations:** 1 Biosafety and Environment Section, Philippine Carabao Center, Science City of Muñoz, Philippines; 2 Production Systems and Nutrition Section, Philippine Carabao Center, Science City of Muñoz, Philippines; 3 Animal Biology Division, Institute of Biological Sciences, College of Arts and Sciences, University of the Philippines Los Baños, College, Los Baños, Philippines; 4 Department of Agriculture, National Livestock Office, Quezon City, Philippines

**Keywords:** *pirB*, protein structure, *Photorhabdus*, *Xenorhabdus*, *Vibrio*

## Abstract

**Objective:**

To compare the structural and functional variations in the Photorhabdus insect-related B* (pirB)* genes of selected bacterial species such as *Photorhabdus luminescens*, *Xenorhabdus doucetiae*, and *Vibrio parahaemolyticus*.

**Materials and Methods:**

The study implemented phylogenetic analysis, three-dimensional (3D) structural modelling, and functional motif analysis of the *pirB* gene of three bacterial genera. Inferred evolutionary relationships as well as functional and structural differences were drawn based on the generated topology of the Neighbor-Joining (NJ) Tree and genetic analysis of protein sequences, domain structures, and functional motifs.

**Results:**

Phylogenetic analysis and NJ tree topology revealed close evolutionary affinity of *Photorhabdus *spp*.* and *Xenorhabdus *spp*.* This is possibly due to their known shared ecological niche as insect pathogens and nematode symbionts. In contrast, the known shrimp pathogen, *V. parahaemolyticus* remarkably showed significant divergence and clustered out from the clade of *Photorhabdus* and *Xenorhabdus*. This can be attributed to the adaptive changes in a marine environment, since *V*.* parahaemolyticus* is a known marine bacterium. The constructed 3D protein structures of *pirB* exhibited conserved transmembrane helices essential for membrane interaction. Species-specific adaptation was also evident in the generated *pirB* 3D model of *V*. *parahaemolyticus*. A unique structural element that confers resistance to environmental stresses was also observed. Analysis of functional motifs depicted evolutionary conservation in membrane interaction domains. Species-specific variations that may reflect adaptations to different host environments and pathogenic strategies were also predominant.

**Conclusion:**

The study provided valuable insights into the structure, function, and evolution of the *pirB* gene of three examined bacterial genera. This can be linked to evolutionary and selective pressures that led to the current pathogenicity of the *pirB* gene, with potential applications in public health, pharmaceuticals, agriculture, and fisheries.

## Introduction

Understanding the intricate and complex role of genetic factors contributing to bacterial pathogenicity is crucial in many fields, such as public health, pharmaceuticals, veterinary medicine, agriculture, and fisheries. In the broadest sense, bacterial pathogenicity can be categorically defined as the capacity of the bacteria to induce and cause infectious diseases in humans, animals, or plants [1]. Therefore, studying and carefully elucidating the mechanisms behind it can lead to increased vaccine efficiency, reduced antimicrobial resistance, production of novel drugs, and improved public health emergency response in general. It may also lead to increased agricultural activity and improved aquaculture production by focusing on bacterial pathogenicity of economically important bacterial pathogens in crops and fish [2–4]. Moreover, a greater understanding of several factors governing it can result in increased capacity in identifying and predicting possible emerging bacterial diseases of public health importance [5,6]. It can also lead to significant advancement towards the general goal of minimizing, if not totally eradicating, the fatal effects of bacterial infections in almost all living organisms, particularly in humans and animals.

At the molecular level, bacteria express various genes that bind to target host cells to facilitate infection, which will later lead to pathogen-induced diseases [7–9]. In this study, we will focus on the Photorhabdus insect-related B (*pirB*) toxin gene, an insecticidal toxin gene first derived from the *W14* genome of the gram-negative enteric bacterium *Photorhabdus luminescens* [10,11]. This gene, which is a part of the *P*. *luminescens* toxin complexes, significantly affects the gravity of pathogenicity of several bacterial species, including *P*. *luminescens *itself*, Xenorhabdus doucetiae*, and *Vibrio parahaemolyticus* [12]. These bacteria have distinct ecological niches and are known to exhibit unique pathogen expression systems, making them important models for closely examining bacterial pathogenicity [13].

The gram-negative bacteria of the genus *Photorhabdus* and *Xenorhabdus* (Enterobacteriaceae) are known as facultative anaerobic insect pathogens and symbionts of nematodes. These motile, rod-shaped, and chemoorganotrophic heterotrophs are characterized by respiratory and fermentative metabolism [14]. They are both known to symbiotically inhabit the intestine of Steinernematidae and Heterorhabditidae, which are among the largest families of entomopathogenic nematodes [15]. They aid nematodes in infecting insect hosts by releasing cascades of toxins, including those encoded by the *pirB* gene. This resulted in the blocking and disruption of the host cell’s regular signaling pathway [16]. Additionally, secondary metabolites with insecticidal and antimicrobial compounds can be synthesized by these bacteria, which further increases their pathogenicity [17].

On the other hand, the gram-negative and halophilic bacterium, *V. parahaemolyticus* (*Vibrionaceae*), naturally occurs and is ubiquitous in both marine and estuarine environments. Unlike *Photorhabdus* and *Xenorhabdus,* which specifically inhabit certain nematode families, *Vibrio* species have a wide host range. It is considered a food-borne pathogen of humans, which can be acquired through consuming undercooked, cross-contaminated, and improperly handled seafood and other marine-derived products [18,19]. This autochthonous and halophilic bacterium can cause varying degrees of illnesses such as acute gastroenteritis, wound infection, and even sepsis [20,21]. In aquaculture, *V. parahaemolyticus *has long been identified as a shrimp pathogen. It is the known causative agent of the deadly Acute Hepatopancreatic Necrosis Disease (AHPND) in shrimp, infecting both the tiger prawn *Penaeus monodon* and the Pacific white shrimp *Litopenaeus vannamei.* In shrimp, the heavily lethal toxin genes, *pirB* together with its homolog *pirA*, target the hepatopancreas, leading to disruption of epithelial cells, tissue necrosis, and massive hemocyte infiltration [22,23]. Moreover, it was also shown that it can cause gut dysbiosis in *P*. *monodon* as revealed by metagenomic analysis of the *16S rRNA *gene [24]. Between these two homologous toxin genes, *pirB* is known to be the main toxin protein that leads to AHPND [25].

Despite the known roles of the *pirB* gene in bacterial pathogenicity, there is a huge knowledge gap in understanding the evolution and adaptation of this gene in several bacterial taxa. Thus far, comparative analysis on structural and functional adaptations of this gene in several bacterial groups, such as *Photorhabdus*, *Xenorhabdus,* and *Vibrio,* is limited or, at worst, lacking. This resulted in an incomplete understanding of the evolutionary pressures and molecular mechanisms that might have shaped the *pirB* gene expression in diverse species. Moreover, foundational knowledge on the adaptations and functional mechanisms of this gene remains largely obscure. Therefore, it is worthwhile to investigate how these adaptations shape and influence pathogenic strategies and microbial infections in both terrestrial insect hosts and marine environments.

Given the important role of this toxin in public health and the aquaculture industry, it is therefore crucial to understand the evolutionary adaptations and functional mechanisms of the *pirB* gene across different bacterial species. Specifically, the goal of the present study is to conduct a comparative structural and functional analysis of the *pirB* gene in *Photorhabdus*, *Xenorhabdus*, and *Vibrio* species and unravel the possible connection between their evolution and adaptation to their pathogenicity. Finally, this study can also provide important data at the molecular and structural level that can help in understanding the bacterial pathogenicity of this highly important gene. 

## Materials and Methods

### Ethical statement

The research is exempted from ethical review under the 2017 Philippine National Ethical Guidelines for Health and Health-related Research due to minimal risk.

### Gene sequences

The *pirB* gene sequences from *Photorhabdus *spp*.*, *Xenorhabdus *spp., and *V. parahaemolyticus* species were downloaded from the National Center for Biotechnology Information (NCBI) database. Sequences were selected based on the established criteria to improve sampling accuracy and to ensure the inclusion of representative strains from diverse ecological backgrounds.

### Phylogenetic analysis

The retrieved sequences were aligned using Clustal Omega as implemented in Molecular Evolutionary Genetics Analysis (MEGA7) software. Neighbor-Joining tree was constructed using the MEGA software, with the Tamura 3-parameter model as a measure of genetic distance. Iteration was set to 1,000 to assess the reliability of the generated tree topology. The evolutionary distances between species were computed to assess the divergence of the *pirB* gene across the tested genera.

### Functional motif analysis

Prediction of functional motifs within the *pirB* proteins was conducted using the Simple Modular Architecture Research Tool (http://smart.embl-heidelberg.de/). Here, conserved domains for membrane interaction and toxin activity, as well as species-specific variations that may give cues to evolutionary adaptations to different host environments, were identified.

### Three-dimensional (3D) structural modeling

Three-dimensional structures of the *pirB* proteins were constructed using the SWISS-MODEL online tool (https://www.expasy.org/resources/swiss-model). Transmembrane regions and functionally important structural elements were emphasized in constructing the model. Model validation was accomplished by comparing available and experimentally determined structures. The impact of species-specific structural differences on protein function was also analyzed.

## Results and Discussion

### Phylogenetic analysis

**Figure 1.**
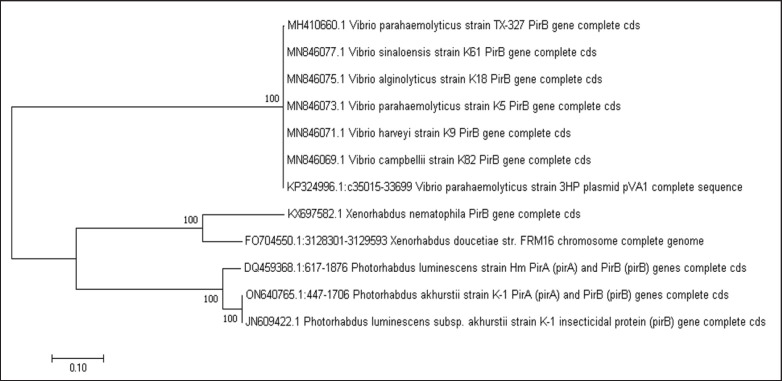
The constructed Neighbor-Joining tree of *pirB* gene sequences from *Photorhabdus*, *Xenorhabdus*, and *Vibrio* species.

The generated neighbor-joining tree of the examined *pirB* gene sequences of *Photorhabdus *spp., *Xenorhabdus *spp., and *Vibrio *spp. suggests divergent evolutionary relationships (Fig. 1). This is likely influenced by distinct selective pressures and adaptations from their respective ecological niches. Among these factors that shaped the functional evolution of *pirB* genes are host specificity, environmental conditions, and immune evasion strategies [26]. Based on the inferred phylogenetic tree, *Photorhabdus *spp. and *Xenorhabdus *spp. exhibited a close evolutionary relationship. These genera of bacteria both belong to the family *Enterobacteriaceae*, and both thrive in a similar environment as insect pathogens and symbionts of nematodes. The close clustering of their *pirB* gene sequences with strong bootstrap support values suggests that these bacteria retained conserved sequences and possibly homologous gene regions because of their shared evolutionary history and similar pathogenic strategies [27,28]. The elucidation of conserved functional motifs and 3D structural features in their *pirB* proteins further strengthens this observation. This reflects the complex yet similar roles these proteins play as they interact with their insect hosts [29].

In contrast, the *pirB* genes of the examined *Vibrio *spp.* (Vibrionaceae)* formed a distinct and separate cluster from the *pirB* genes of *Photorhabdus* and *Xenorhabdus*, similar to the result of Hao et al. [30]. The clade of the *pirB* gene sequence of *Photorhabdus* and *Xenorhabdus* was basal to the clade of *Vibrio*, which also showed a strong bootstrap support value. This result indicates a significant divergence of *Vibrio *from *Photorhabdus* and *Xenorhabdus*, which is consistent with the known evolutionary separation of *Vibrio* species from the enterobacteria. This divergence was also observed in the constructed model, which yielded unique structural features and functional motifs in the *Vibrio* *pirB* protein. The recognizable capability of *V*. *parahaemolyticus *to infect a wide host range can be attributed to its molecular adaptation, genetic evolution, and biochemical changes during survival in marine environments [12]. This unique capability to infect both humans and animals is likely due to the acquisition of unique virulence factors, possibly due to horizontal gene transfer, as seen in the distinct structural elements of the *pirB* protein that are absent in *Photorhabdus* or *Xenorhabdus*.

The result of phylogenetic analysis highlights the evolutionary relationship of *pirB* genes between the tested bacterial groups. The close evolutionary relationship of *Photorhabdus* and *Xenorhabdus* suggests that their *pirB* genes might have evolved under similar selective and adaptive pressures, resulting in conserved functions and homologous genes relative to their symbiotic and pathogenic interactions with their insect hosts. In contrast, the divergence of the *Vibrio parahaemolyticus pirB* gene reflects different niche adaptations resulting in different gene assemblies and divergent evolution. The unique adaptations exhibited in the *Vibrio* *pirB* proteins highlighted the role of environmental factors and host diversity in driving the evolution of bacterial virulence genes [10].

### Three-dimensional protein structure

**Figure 2.**
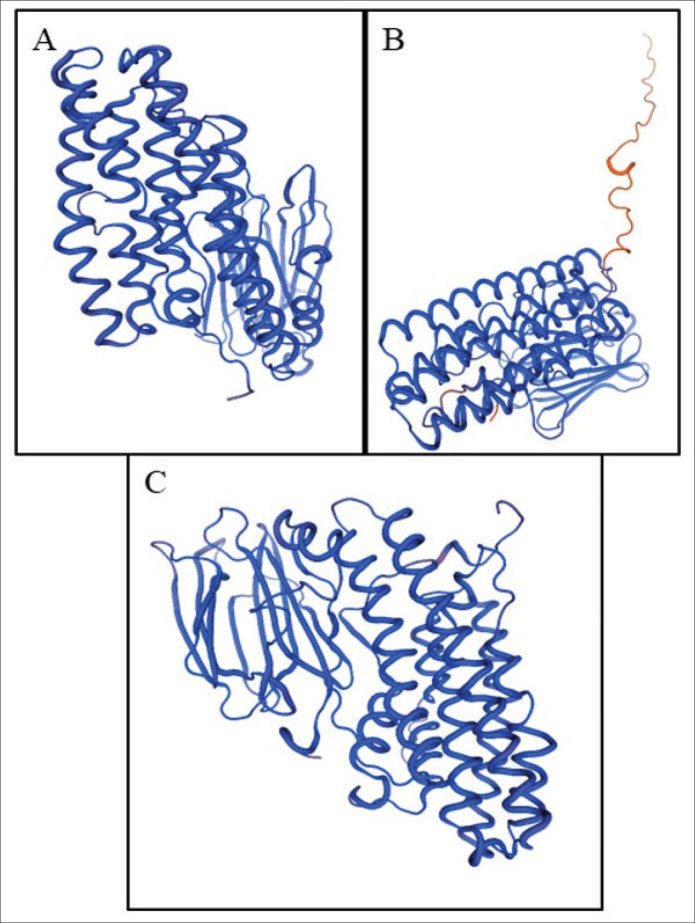
Three-dimensional (3D) models of *pirB *protein structures in *Photorhabdus luminescens* (A), *Xenorhabdus doucetiae* (B), and *Vibrio parahaemolyticus* (C).

The three-dimensional structures (Fig. 2) of the *pirB* proteins in *P. luminescens*, *X. doucetiae*, and *V. parahaemolyticus* reveal conserved elements and species-specific adaptations important for their pathogenicity [5].

The 3D structure of the *pirB* protein in *P. luminescens* (Fig. 2A) is characterized by visible multiple transmembrane helices linked by hydrophobic amino acid residues. The structural differences observed in the *PirB* proteins of these bacteria have a significant role in inferring their toxicity. These variations have a direct influence on their capability to efficiently penetrate host cell membranes, toxin binding affinity, and resistance to environmental stressors. These helices are integral to the protein’s ability to inject toxin into host cell membranes, facilitating the transfer of toxic elements into the host cells. Another notable characteristic of the protein structure is the flexible loop regions and beta-sheet domains. These are directly involved in its pathogenicity by disrupting the host cell through cell-to-cell interactions with host cell receptors and with other bacterial proteins [10].

The generated *pirB* 3D structures of *X. doucetiae *(Fig. 2B) and *P. luminescens* have both conserved transmembrane helices for membrane association. The seen structural variations in the surface-exposed loops suggest adaptations that allow the interaction of *Xenorhabdus* with host cells and for evasion of host immune responses. These structural differences have a distinct function of improving the protein’s permeability in the host cell, such as within the insect hosts or within the cells of symbiotic nematodes.

**Figure 3.**
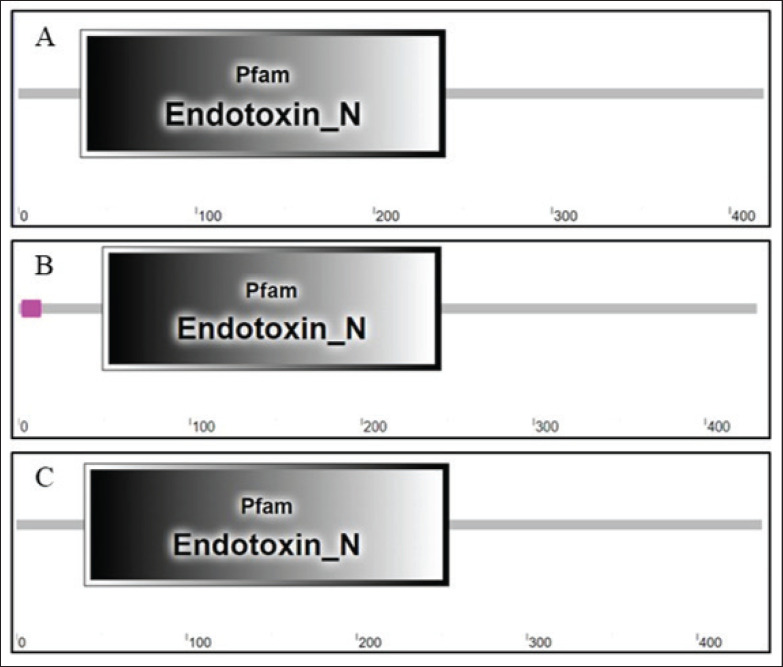
Predicted functional domains of *pirB* in (A) *Photorhabdus luminescens*, (B) *Xenorhabdus doucetiae*, and (C) *Vibrio parahaemolyticus*.

The 3D structure of the *pirB* protein in *V. parahaemolyticus* (Fig. 3C) is divergent in relation to the other two species. It can be deduced that the overall architecture of the *Vibrio* *pirB* protein has an additional alpha-helix and beta-sheets, which is a unique structural feature only observed in *V*. *parahaemolyticus.* The core transmembrane regions are also conserved for the increased interaction with host membranes. These characteristics reflect the adaptations necessary for *Vibrio* to survive in marine environments and infect a broader range of hosts. These unique structural elements are also helpful during stringent and harsh environmental conditions such as high salinity and fluctuating temperatures. Moreover, it may also facilitate interactions with host molecules different from those exhibited by *Photorhabdus* and *Xenorhabdus*.

### Functional motifs

The analysis of *pirB* proteins’ functional motifs of *P. luminescens*, *X. doucetiae*, and *V. parahaemolyticus* (Fig. 3) highlights the functional roles of these proteins in bacterial pathogenicity.

The *pirB* protein in *P. luminescens* has conserved functional motifs for efficient pathogenesis [1]. These include hydrophobic regions resulting in the formation of transmembrane domains. It enables better permeability of protein to host cell membranes, a key step in initiating its toxic effects. Additionally, motifs with flexible loops were evident. These are active sites rich in amino acids, such as serine, alanine, and glycine, that can bind to host cell receptors or can catalyze enzymatic reactions that disrupt host cell functions.

**Figure 4.**
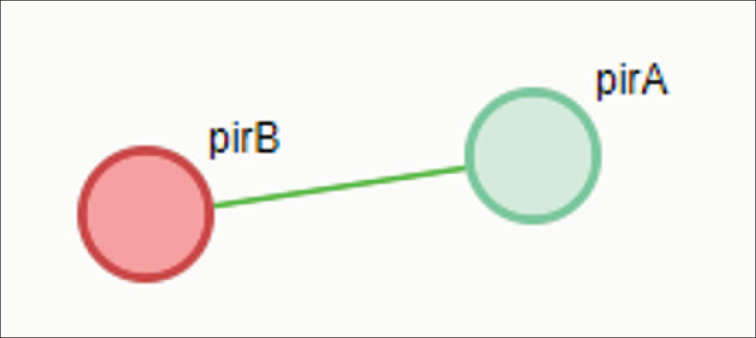
Predicted functional partnership between *pirB* and *pirA* observed in *Xenorhabdus* sp.

Note: Green line denotes gene neighborhood.

In *X. doucetiae*, the *pirB* protein shares several motifs found in *P. luminescens*, particularly those related to membrane interaction. These conserved motifs are important in bacterial pathogenicity, specifically in the protein’s ability to associate with host cell membranes and in delivering toxic effects. The presence of hydrophilic regions that are species-specific variations indicates adaptations to either different host environments or pathogenic strategies. These variations can directly influence the protein’s solubility, stability, and interaction with other proteins, allowing *Xenorhabdus* to fit its pathogenic mechanisms depending on the ecological niche. Surprisingly, *X. doucetiae *is the lone bacterial species that exhibited a relationship between the binary toxins *pirA* and *pirB *(Fig. 4). This functional partnership between *pirB* and *pirA* is an intriguing aspect of this bacterial species’ pathogenic strategy. These *pirA* and *pirB* are components of a binary toxin system. They mutually work together to infer their toxic effects on the host organism. In *X. doucetiae*, the *pirB* gene functions in conjunction with *pirA*, forming a synergistic partnership that enhances the bacterium’s ability to infect and later cause disease to the host organism. In this synergistic work, *pirA* facilitates the binding of the toxin complex to the host cell membrane, allowing *pirB* to penetrate the cell and disrupt vital cellular processes. This binary toxin system precisely targets the host cell, making it a powerful tool for *Xenorhabdus* in overcoming the host defense system.

The *pirB* protein in *V. parahaemolyticus* exhibits a higher degree of divergence in its functional motifs compared to other examined species. It retains hydrophobic regions for membrane interaction, but the specific sequence within these motifs has considerable variations. This observed divergence is a possible adaptation of *Vibrio* to a marine environment, as well as its interactions with various hosts. The *Vibrio* *pirB* protein also has possible novel functional domains not present in the other species. This is potentially related to specific virulence factors or environmental adaptations, including but not limited to resistance to high salinity or the ability to infect marine organisms.

The result of this study can be used for biocontrol and disease prevention strategies. Particularly, the understanding of *pirB* functional adaptations can contribute to the development of novel antimicrobial drugs (including engineered bacterial strains for targeted pest control), public health response, and identifying vaccine candidates for disease management in fish farms.

This study utilized computational modeling and *in-silico* sequence analysis; hence, experimental validation through mutagenesis or functional assays is needed to support the predicted structural and functional differences. The study may not also represent the full diversity of *pirB* genes in all bacterial genera since the examined sequences were limited to publicly available gene sequences of bacterial groups of particular interest.

## Conclusion

The 3D protein structures and functional motifs of the *pirB* gene products from *P. luminescens*, *X. doucetiae*, and *V. parahaemolyticus *reveal important insights into evolution and species-specific adaptations. The conserved elements in transmembrane regions and functional motifs involved in membrane interaction suggest the crucial role of these proteins in bacterial pathogenicity. Meanwhile, the structural and functional divergences observed in *Vibrio* denote evolutionary and selective pressures that led to adaptation in unique ecological niches as well as the regulation of pathogenic strategies of each species. Understanding these structural and functional aspects delivers important insights into the molecular mechanisms of bacterial virulence and the evolutionary dynamics of pathogenic genes, such as *pirB*. Finally, given the importance of *pirB* genes in bacterial pathogenicity, it is therefore prudent to study and understand both the functional and structural differences within different bacterial groups to be used as a potential baseline study in public health, agriculture, pharmaceutical, and fisheries studies in the future.

## List of abbreviations

AHPND, Acute Hepatopancreatic Necrosis Disease; PDB, Protein Data Bank;* pirA*, *Photorhabdus* insect-related toxin A; *pirB*, *Photorhabdus* insect-related toxin B; MEGA7, Molecular Evolutionary Genetics Analysis 7 (software); NCBI, National Center for Biotechnology Information; SMART, Simple Modular Architecture Research Tool (for functional motif analysis); 3D, Three-Dimensional(used for structural references).
